# Unraveling Osteoarthritis: Mechanistic Insights and Emerging Therapies Targeting Pain and Inflammation

**DOI:** 10.3390/biom15060874

**Published:** 2025-06-16

**Authors:** Muskan Alad, Fajer Yousef, Laura M. Epure, Angelina Lui, Michael P. Grant, Geraldine Merle, Nicoletta Eliopoulos, Jake Barralet, John Antoniou, Fackson Mwale

**Affiliations:** 1 Department of Surgical and Interventional Sciences, Faculty of Medicine and Health Sciences, McGill University, Montreal, QC H3T 1E2, Canadalaura.epure@mcgill.ca (L.M.E.);; 2 Orthopaedic Research Laboratory, Lady Davis Institute for Medical Research, McGill University, Montreal, QC H3T 1E2, Canada; 3 Orthopedic Department, SMBD-Jewish General Hospital, McGill University, Montreal, QC H3T 1E2, Canada; 4 Chemical Engineering Department, Polytechnique Montréal, Montreal, QC H3T 0A3, Canada; 5 Lady Davis Institute for Medical Research, McGill University, Montreal, QC H3T 1E2, Canada; 6 Faculty of Dentistry, McGill University, Montreal, QC H3T 1E2, Canada

**Keywords:** osteoarthritis, pain, cartilage, degeneration, inflammation, regeneration, pain management

## Abstract

Osteoarthritis (OA) is now widely recognized not merely as a cartilage-centric disease but as a multifactorial disorder affecting the entire joint as an organ, including the articular cartilage, subchondral bone, synovium, ligaments, menisci, and the innervating neural elements. This review explores the complex pathophysiology of OA with a focus on the emerging mechanisms of pain and inflammation that extend beyond articular cartilage degradation. Joint inflammation driven by immune activation in response to cellular stress signals promotes the release of pro-inflammatory mediators and catabolic enzymes. Key signaling pathways such as NF-κB, MAPKs, and JAK/STAT amplify these responses, and pain is sustained through peripheral and central sensitization, contributing to exacerbating symptoms even in the absence of visible joint damage. This review also integrates molecular and cellular mechanisms to highlight innovative therapies aimed at modifying both the structural damage and neurosensory drivers of pain. These approaches offer the potential to not only alleviate symptoms but also alter disease progression, signaling a move toward personalized, mechanism-based treatments. Given the intricate interactions among joint tissues, immune activation, and sensory processing, a comprehensive strategy that targets both structural degeneration and neuroinflammation is essential for the future of OA management. Emphasizing the joint as an integrated organ, we advocate for translational research linking molecular pathology with clinically meaningful outcomes.

## 1. Pathophysiology of OA as a Whole-Joint Disease

Osteoarthritis (OA) is a worldwide joint disorder that plays a major role in the global chronic pain scenario [1,2,3,4]. It progresses through distinct stages, each marked by an increasing severity of symptoms and joint degeneration, leading to pain and disability. The effectiveness of current pain management strategies for OA is limited, as traditional painkillers often do not offer sustained relief and are linked with adverse side effects [5,6,7,8,9,10]. This gap in effective pain management often leads to joint replacement, which accounts for 90% of all hip and knee arthroplasties, imposing a significant economic burden [11,12,13,14]. The annual cost in the United States is estimated at USD 136.8 billion, including both healthcare expenses and indirect costs [15,16,17].

**Figure 1:**
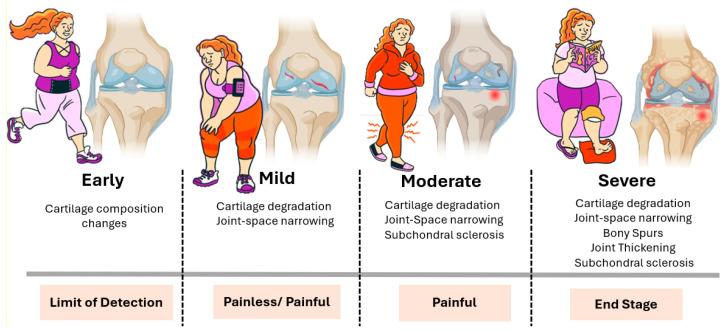
Clinical and structural progression of osteoarthritis. Illustration of the typical stages of OA progression, both in terms of joint structure and patient experience. In the early stage, subtle biochemical and compositional changes occur in the cartilage, typically without noticeable symptoms and below the threshold of standard detection methods. In the mild stage, cartilage degradation and joint space narrowing become evident, although symptoms may range from absent to intermittent pain. The moderate stage is characterized by more pronounced cartilage loss, subchondral bone sclerosis, and persistent joint pain. In the severe stage, structural damage is extensive, including bony spur formation (osteophytes), joint thickening, and significant subchondral sclerosis. This stage is typically associated with chronic pain and functional impairment and often represents the endpoint of disease progression. The horizontal bars below each stage indicate both the clinical detectability and pain profile, underscoring the disconnect between the radiographic findings and patient-reported symptoms at earlier stages.

Traditionally regarded as a disease primarily affecting articular cartilage, OA is now widely understood to involve the entire joint as an organ system [18]. This paradigm shift recognizes that OA pathology includes not only cartilage degradation but also synovial inflammation, subchondral bone remodeling, meniscal damage, ligament laxity, and alterations in the periarticular muscles, with each joint tissue playing a distinct role in the pain and dysfunction associated with the disease (Figure 1) [18,19,20,21,22,23].

**Figure 2:**
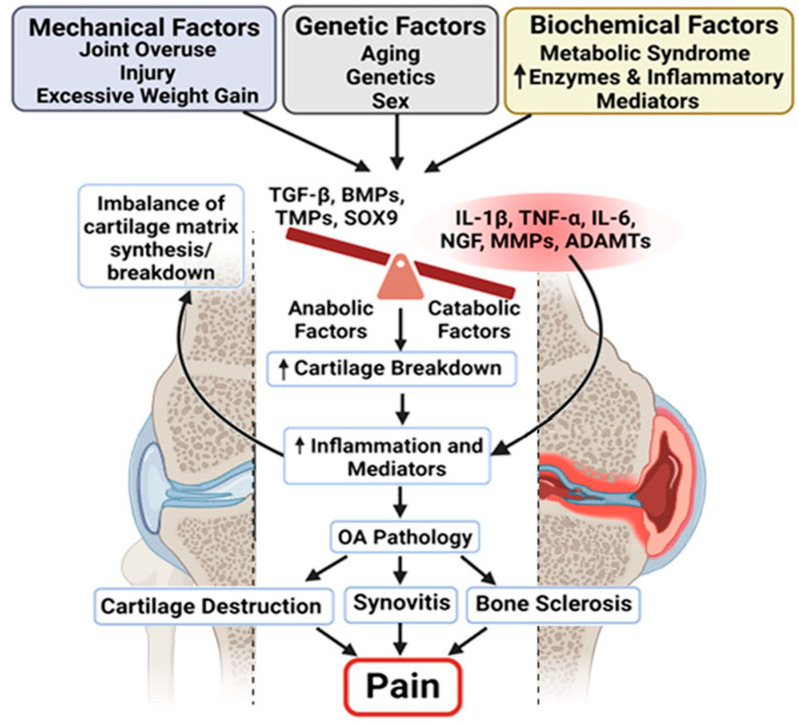
Multifactorial drivers of osteoarthritis pathology and pain. Schematic illustrating the complex interplay between mechanical, genetic, and biochemical factors in the development and progression of OA. Mechanical stressors (e.g., joint overuse, injury, excess weight), genetic predispositions (e.g., age, sex, inherited traits), and biochemical influences (e.g., metabolic syndrome and inflammatory mediators) converge to disrupt the balance between anabolic and catabolic processes in the joint. This imbalance leads to an increased breakdown of the cartilage matrix and the heightened production of inflammatory mediators. The resulting inflammation contributes to the key pathological features of OA: cartilage destruction, synovitis, and subchondral bone sclerosis. Collectively, these changes lead to chronic joint pain, the hallmark symptom of OA, highlighting the need for therapies that address both structural damage and inflammatory pain pathways. The arrows indicate proposed mechanistic links contributing to the progression of joint tissue degeneration and pain.

An imbalance between anabolic and catabolic activities in chondrocytes leads to the deterioration of articular cartilage, which cushions the ends of bones [24,25], resulting in pain and reduced joint function [18,19,25,26,27]. Changes in the subchondral bone, such as the formation of bone spurs and structural alterations, can further worsen symptoms [27,28,29,30,31,32,33,34]. The inflammation of the synovial membrane contributes to swelling and discomfort, while damage to the surrounding ligaments and muscles can compromise joint stability and function, intensifying OA symptoms [32,33,35,36,37,38,39,40]. These pathological changes seen in OA are due to a combination of mechanical, genetic, and biochemical processes that occur in these joint tissues (Figure 2) [22,25,26,41,42]. Biochemical processes involve the release of enzymes and inflammatory mediators that further damage the joint structure [43]. Inflammation plays a central role in OA, leading to pain, swelling, and further tissue degradation [19,26,43,44,45,46]. Mechanical stress from overuse, injury, or excess weight can lead to cartilage breakdown and changes in the bone, contributing to the development and progression of OA [25,47,48,49,50]. The interaction between these factors leads to a cycle of worsening joint damage and symptoms [25,49].

Therefore, understanding the individual contributions and the complex, multifactorial interplay of joint tissues in relation to pain is essential not only for comprehending symptomatic OA but also for developing effective treatments that target the multifaceted nature of OA [44,51,52,53]. This broader understanding significantly influences drug development approaches, as effective treatments must reach multiple joint compartments and cell types. Moreover, outcome measures should extend beyond cartilage repair to encompass overall joint health and functional improvement.

## 2. Pain as Both a Sensory and Emotional Experience

Nociception is the physiological process of detecting harmful stimuli and forms the basis of pain experience [54,55,56]. Upon encountering potentially damaging stimuli, the nociceptors, which are sensory neurons in the tissues, initiate pain transmission [54,55,56,57,58]. This process is intricately linked to aberrant inputs from dorsal root ganglion (DRG) neurons. These pseudo-unipolar cells possess an axonal stalk that branches into two terminals: the peripheral terminal, which innervates peripheral tissues, and the central terminal, which extends to the dorsal horn of the spinal cord [59]. This unique structure enables nociceptors to both receive and transmit signals at each terminal. Triggered by a spectrum of stimuli detected by specialized receptors, they initiate a cascade of voltage-gated sodium and potassium channels, instrumental in the generation and propagation of action potentials [59,60,61]. These action potentials convey distress signals along the nociceptor axons, ultimately transmitting them to higher levels of the nervous system, from the brainstem to the thalamus and cortex [62].

Unlike nociception, pain is shaped by biological, psychological, and social factors, making it a subjective experience not always reflected in nerve activity. Pain also plays a fundamental role in the healing process, serving as an alert mechanism that initiates behaviors promoting recovery following surgery, injury, or illness [63]. The mindset of a patient can significantly impact their healing process. Based on the opinion of Stanford experts, positive attitudes and supportive social environments have been shown to measurably enhance physical healing [64]. This indicates that the perception and internal processing of pain are integral to recovery. Effective pain management can improve quality of life and decrease neuroplastic changes associated with pain [65,66].

## 3. Exploring Pain in OA: Beyond Sensation to Emotion

Nociceptors are abundant in the joint capsule, ligaments, periosteum, menisci, subchondral bone, and synovium, playing a key role in the complex process of perception [21,67,68,69]. Although cartilage lacks nerve structures and does not directly cause pain, its degradation or damage releases factors that stimulate pain [69,70,71]. Moreover, the role of subchondral bone is increasingly recognized in the pathogenesis of OA pain [23,72]. Changes in bone structure and increased bone turnover can contribute to the pain experienced by OA patients [71,73]. The subchondral bone might become more sensitive due to an influx in nerve fibers or changes in blood supply, leading to an enhanced pain response [28,74,75,76]. However, the precise mechanisms and specific joint tissues responsible remain unclear [34,77]. A comprehensive review by Martel-Pelletier et al. provides an in-depth analysis of OA, including its pathophysiology, structural changes within joints, and the multifactorial nature of pain in OA. The authors discuss the complex interplay between cartilage degradation, inflammation, and the involvement of other joint tissues. This reference is valuable as it offers insights into the pain mechanisms, highlighting the areas where further research is needed to effectively treat this condition [41].

**Figure 3:**
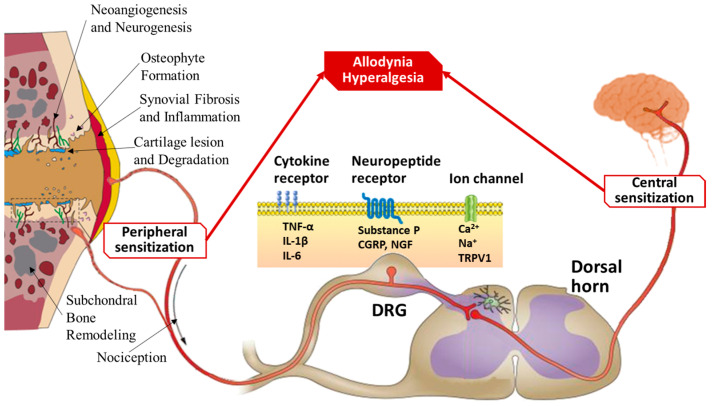
Mechanisms of pain sensitization in osteoarthritis. Schematic illustrating the molecular and neural pathways contributing to OA-associated pain, with a focus on peripheral and central sensitization processes. Joint degeneration triggers peripheral sensitization through cartilage degradation, subchondral bone remodeling, osteophyte formation, synovial inflammation, and neoangiogenesis, leading to increased nociceptive signaling. Inflammatory mediators such as TNF-α, IL-1β, and IL-6 bind to cytokine receptors on nociceptive neurons. Neuropeptides such as substance P, CGRP, and NGF act on their respective receptors, while ion channels (e.g., TRPV1) facilitate calcium and sodium influx, amplifying pain signals. These changes culminate in allodynia (pain due to non-painful stimuli) and hyperalgesia (increased pain from painful stimuli). Persistent nociceptive input from the periphery to the DRG and dorsal horn of the spinal cord leads to central sensitization, whereby the central nervous system becomes hyperresponsive to sensory input, further contributing to chronic pain in OA.

Under normal circumstances, nociceptive responses to painful stimuli are transient. Adding to the complexity of this pain pathway, any disturbances in the biochemical environment of the joint, mediated by factors such as cytokines and neuropeptides, can lower the activation threshold of nociceptors, leading to heightened sensitivity and pain [78,79,80,81]. Therefore, in the context of chronic pathology, pain pathways can undergo significant changes, leading to hypersensitivity, a state known as sensitization. This increased sensitivity can lead to mechanical allodynia, causing pain during joint movement (Figure 3) [58,65,82]. Furthermore, the continued input from pain sensors can induce long-lasting changes in the central nervous system, resulting in central sensitization, characterized by the prolonged hyperexcitability of the pain pathway [57,58,81,83,84].

Researchers are exploring the concept of central sensitization, where the central nervous system becomes more sensitized to pain signals from the joint, amplifying the perception of pain [58,65,83,85,86,87]. Central sensitization may disrupt the correlation between structural joint changes and pain perception, explaining why some individuals with OA experience pain that appears disproportionate to radiographic findings [65,88]. Interestingly, the effects of central sensitization may be reversible after successful joint replacement surgeries, suggesting that ongoing peripheral input is necessary to sustain central nervous system alterations in most patients [65,89].

### 3.1. The Role of DAMPs and PRRs in the Pathogenesis of Pain in OA

OA is frequently referred to as a “wear and tear” disease [18,19,26,90,91,92,93,94]. When cartilage is damaged, it releases Damage-Associated Molecular Patterns (DAMPs), which are diverse and include extracellular matrix (ECM) breakdown products, such as fibronectin fragments, and cellular alarmins like high-mobility group box 1 (HMGB1), S100A8/9, and HSP [95,96]. These molecules are not merely debris; they actively participate in the disease process, leading to a cascade of molecular responses that perpetuate joint degeneration [19,34,70,97,98,99,100,101]. They function as endogenous danger signals, alerting the body to tissue damage [95,102,103,104,105,106,107].

**Figure 4:**
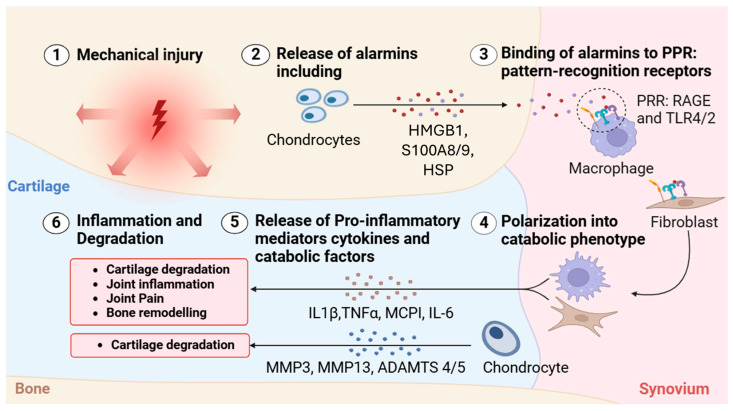
Alarmin-mediated activation of inflammatory cascades in osteoarthritis. Schematic depicting the sequential molecular events initiated by mechanical injury that contribute to inflammation and tissue degradation in OA. (1) Mechanical injury to joint tissues leads to cellular stress and damage, particularly within cartilage. (2) Chondrocytes respond by releasing DAMPs, also known as alarmins, including high-mobility group box 1 (HMGB1), S100A8/9, and heat shock proteins (HSPs). (3) These alarmins bind to pattern-recognition receptors (PRRs), such as RAGE and Toll-like receptors (TLR2/4), primarily on macrophages. (4) This interaction activates fibroblasts and chondrocytes, polarizing them into a catabolic phenotype. (5) Polarized cells release a range of pro-inflammatory cytokines and catabolic enzymes, including IL-1β, TNF-α, monocyte chemoattractant protein-1 (MCP1), IL-6, matrix metalloproteinases (MMP-3 and MMP-13), and aggrecanases (ADAMTS-4 and -5). (6) These mediators drive a feedback loop of inflammation, cartilage degradation, joint pain, and bone remodeling, perpetuating the progression of OA.

DAMPs are recognized by pattern-recognition receptors (PRRs), which are expressed by various cells within the joint, including synovial macrophages, fibroblast-like synoviocytes, and chondrocytes (Figure 4) [34,95,108,109,110,111,112,113,114]. Upon DAMP recognition, PRRs trigger intracellular signaling pathways that lead to the production of inflammatory mediators such as cytokines (e.g., IL-1β and TNF-α), chemokines, and other factors that promote inflammation and pain. The binding of alarmins to PRRs initiates a cascade of inflammatory responses that contribute to joint damage [96,115,116]. The activation of PRRs leads to the upregulation of proteolytic enzymes, including matrix metalloproteinases (MMPs) and aggrecanases (ADAMTSs), which further degrade the ECM of the cartilage [44,91,108,115,117,118,119,120]. This production of inflammatory mediators and proteolytic enzymes establishes a feedback loop. As the ECM degrades, more DAMPs are released, which in turn activate more PRRs, leading to further inflammation and tissue damage. This cycle becomes self-amplifying and is a central driver of the progressive nature of OA [18,19,26,96,98,99,121].

### 3.2. Pain Markers in Osteoarthritis

Pain in OA is multifactorial, arising from the interplay between cartilage degradation, inflammatory mediators, and nociceptive signaling [34,122]. In response to cartilage damage, chondrocytes attempt to repair the tissue by increasing ECM production [123]. However, this process also triggers the production of catabolic factors like Runx2 and matrix metalloproteinase-13 (MMP-13), which break down ECM even further [120,124]. This leads to the early stages of OA marked by cartilage thinning, type X collagen production, and the formation of surface fibrillation [125,126].

The DAMPs released due to the breakdown of the cartilage trigger inflammatory signaling cascades, with IL-1β and tumor necrosis factor-alpha (TNF-α) as primary pro-inflammatory cytokines implicated in OA [18,96,127]. IL-1 receptor type I (IL-1RI), activated by the binding of IL-1β, promotes the upregulation of aggrecanases, such as A Disintegrin and Metalloproteinase with Thrombospondin Motifs (ADAMTS-4 and ADAMTS-5) and MMPs, which further degrade ECM components [18,120,127,128,129]. Studies have shown that OA chondrocytes express increased levels of IL-1RI compared to normal chondrocytes [130]. Additionally, elevated levels of IL-1β have been detected in OA-affected synovial fluid, cartilage, and subchondral bone [131,132]. Alongside IL-1β, TNF-α plays a synergistic role by activating TNF receptors (TNF-Rs), which further amplify inflammation and ECM degradation [133,134,135].

**Figure 5:**
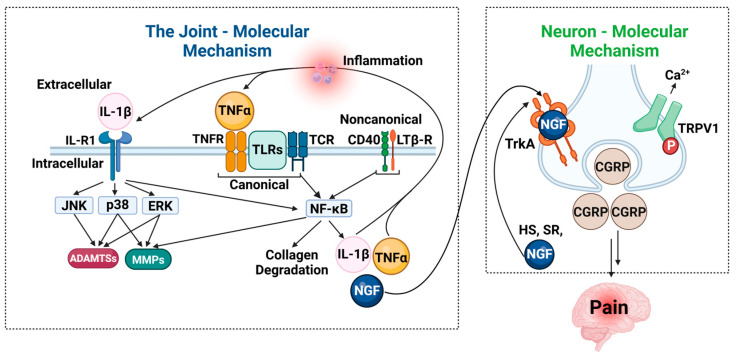
Molecular mechanisms linking inflammation and pain in osteoarthritis. Schematic outlining the key molecular pathways contributing to inflammation and pain in osteoarthritis (OA), divided into joint-related and neuron-related mechanisms. **Left panel**—Joint: molecular mechanism: inflammatory cytokines such as IL-1β and TNF-α bind to their respective receptors (IL-1R and TNFR), initiating intracellular signaling cascades through the canonical and non-canonical NF-κB pathways. These cascades activate downstream kinases such as JNK, p38, and ERK, resulting in upregulation of matrix-degrading enzymes, including matrix metalloproteinases (MMPs) and aggrecanases (ADAMTSs). This contributes to extracellular matrix (ECM) degradation, synovial inflammation, and joint degeneration. Additional signaling via TLRs, CD40, and the lymphotoxin-β receptor (LTβR) reinforces the inflammatory response. These processes also stimulate the production of NGF, IL-1β, and TNF-α, further propagating inflammation. **Right panel**—Neuron: molecular mechanism: NGF binds to its high-affinity receptor TrkA on sensory neurons, triggering retrograde transport and the release of pain mediators such as calcitonin gene-related peptide (CGRP). NGF-TrkA signaling also activates ion channels like TRPV1, increasing calcium influx and neuronal excitability, which contributes to peripheral sensitization and pain perception. Together, these molecular interactions underscore the bidirectional relationship between joint inflammation and neuronal pain mechanisms in OA.

At the molecular level, IL-1β and TNF-α activate several key signaling pathways that cause cartilage degradation and pain in OA [18]. The mitogen-activated protein kinase (MAPK) pathway is one of them and includes Janus Kinase/Signal Transduction and Activators of Transcription (JNK/STAT), p38, and Extracellular Signal-Regulated Kinase (Figure 5) [18,136,137,138]. These pathways regulate the expression of matrix proteins and catabolic enzymes, such as MMP-1, -3, -9, -13, and ADAMTS-4 and -5, with the release of inflammatory cytokines (IL-1β, IL-6, IL-17, IL-23, CCL2) and pain mediators, including the nerve growth factor (NGF) and substance P (SP) [18,138,139].

Another important pathway that plays a central role in cartilage degradation and inflammation is the nuclear factor kappa B (NF-κB) [140,141,142,143,144,145]. NF-κB signaling can occur through two main mechanisms: the canonical NF-κB pathway, triggered by Toll-like receptors (TLRs), TNF receptors (TNF-Rs), and IL-1RI, and the non-canonical pathway, driven by CD40, lymphotoxin β (LTβ), and B-cell activating factors [143,144,145]. The exacerbation of these inflammatory mediators further sensitizes nociceptive pathways. IL-1β and TNF-α induce the expression of the nerve growth factor (NGF), which binds to tropomyosin receptor kinase A (TrkA) on sensory neurons [146,147]. This NGF–TrkA complex is retrogradely transported to neuronal cell bodies, upregulating pain-related gene expression, including the synthesis of substance P (SP) and the calcitonin gene-related peptide (CGRP), which enhance pain transmission [146]. Furthermore, NGF binding to TrkA activates ion channels such as transient receptor potential vanilloid 1 (TRPV1) and voltage-gated sodium channels, leading to nociceptor depolarization and sensitization [146,148,149]. Elevated NGF and SP levels have been detected in OA joints, further reinforcing their role in chronic pain development [150,151,152,153].

Another emerging aspect of inflammation in OA pathophysiology is macrophage polarization, which occurs through cellular crosstalk [114,135,154]. In response to microenvironmental stimuli, macrophages differentiate into classically activated (M1) macrophages or alternatively activated (M2) macrophages [155,156]. M1 macrophages play a dominant role in driving inflammation and ECM breakdown, while M2 is involved in tissue remodeling and immunomodulation [127,155,156]. An imbalance favoring M1 macrophages driven by IL-1β and TNF-α leads to the further secretion of pro-inflammatory mediators, resulting in persistent inflammation, worsening cartilage degradation, and chronic pain in OA [127,154,155,156].

The synovial membrane also undergoes chronic inflammatory changes, involving both innate and adaptive immune responses [18,44,154]. The continued release of inflammatory mediators amplifies pain signaling, accelerating the disease progression. Additionally, mast cell activation leads to the secretion of histamine, serotonin, and additional NGF, creating a positive feedback loop that perpetuates inflammation and pain [157,158].

## 4. Current Treatments for OA

**Figure 6:**
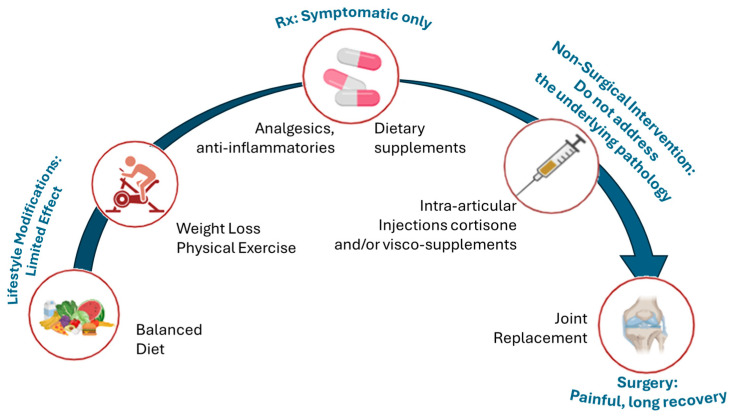
Current stepwise management of OA and associated limitations. Figure outlining the typical progression of treatment strategies for OA, from early lifestyle modifications to end-stage surgical intervention. The current treatment approach for osteoarthritis (OA) begins with lifestyle changes and non-opioid medications to manage symptoms but does not address the root cause of the disease. As OA progresses, options like intra-articular injections may provide temporary relief, but cartilage degeneration continues. When these methods fail, joint replacement surgery becomes necessary, though it is invasive and costly. This progression underscores the urgent need for therapies that can modify the disease earlier and more effectively.

The current treatment options for OA are primarily palliative, as no established gold standard for disease-modifying therapy exists [159]. The non-pharmacological interventions are the foundation of early OA therapy (Figure 6). Weight loss, exercise, and physical therapy can significantly reduce pain and improve mobility in many patients [160].

Furthermore, pharmacological treatments such as Acetaminophen and oral non-steroidal anti-inflammatory drugs (NSAIDs) are commonly used to reduce pain and inflammation [161,162]. However, the long-term use of these medications can cause gastrointestinal, renal, or cardiovascular side effects [162,163]. In addition, topical analgesic therapies offer a non-invasive option with fewer systemic side effects. These include capsaicin cream, which depletes substance P from sensory neurons to reduce pain signaling, and methyl salicylate creams, which produce a counterirritant effect and mild anti-inflammatory action. Such topical agents can be particularly beneficial for patients with localized joint pain or those who cannot tolerate systemic medications [164]. In patients with severe pain, unresponsive to first-line analgesics, the short-term use of opioids or adjuncts like duloxetine may be considered, but these carry significant side effects. While these medications can improve day-to-day comfort, they do not prevent ongoing cartilage degeneration [162,165].

For localized symptomatic relief, intra-articular (IA) injections are another key option (Figure 6). Corticosteroid injections can rapidly reduce joint inflammation and pain, with a temporary effect [165]. However, repeated steroid injections are generally limited due to potential deleterious effects on cartilage, such as chondrocyte toxicity, accelerated cartilage thinning, subchondral bone osteonecrosis, and risks of soft-tissue atrophy or systemic glucocorticoid exposure [165,166,167,168]. Visco supplementation with hyaluronic acid is also used in knee OA to improve lubrication; however, it may cause transient adverse events, including injection-site pain, swelling or effusion, local erythema, allergic reactions, and acute pseudoseptic inflammatory flares [169,170].

Surgical interventions such as joint arthroplasties become appropriate in advanced OA (Figure 6) or when conservative measures fail to provide relief [171,172,173]. Although they can reduce pain and restore joint function, it remains an invasive, high-cost surgery typically reserved for late-stage disease [171]. Table 1 summarizes the spectrum of current management options for OA [23,162,165].

## 5. Regenerative Therapies Targeting Pain Pathways

Given the limitations of conventional therapies, there is a need for alternative approaches that not only slow disease progression but also effectively alleviate pain [164]. Tissue engineering and regenerative medicine have therefore emerged as promising strategies to promote the growth of new cartilage, protect existing tissue, and potentially reverse the pathological joint environment, thereby reducing pain [164,174,175,176]. Since chronic inflammation is a key driver of both joint degeneration and pain, incorporating anti-inflammatory strategies within regenerative approaches is essential [124]. These strategies integrate engineering techniques with biological components, such as cells and bioactive molecules, to rebuild functional tissue and provide more lasting relief from OA-related pain [177,178].

### 5.1. Orthobiologics in Osteoarthritis: PRP, Cell Therapy, and Tissue Engineering Approaches

A variety of orthobiologic therapies have emerged as promising interventions for OA. Orthobiologics refer to a broad class of biological products used to promote musculoskeletal healing and include platelet-rich plasma (PRP) and its alternatives, bone marrow aspirate concentrate (BMAC), bone grafts and matrix substitutes, bone morphogenic proteins (BMPs), mesenchymal stem cells (MSCs), growth factors, and tissue engineering approaches using natural, synthetic, or composite scaffolds [179]. Among these, PRP and MSCs have garnered substantial attention for their regenerative and anti-inflammatory effects in OA. PRP therapy uses a growth factor-rich biologic to promote tissue repair and modulate inflammation in the joint microenvironment [180]. In contrast, stem cells, particularly MSCs, are undifferentiated cells that, when stimulated, can develop into specialized tissue cells capable of replacing worn or damaged tissue [177]. It is important to distinguish that MSC administration alone constitutes a form of cell therapy rather than tissue engineering, which, by definition, requires the integration of cells, scaffolds, and growth factors [177]. The inclusion of biomaterial scaffolds with MSCs would then appropriately qualify under tissue engineering [178,181]. Despite their different mechanisms, both therapies target both cartilage structural degeneration and chronic inflammation, thereby providing OA-related pain relief [182,183,184].

PRP is a blood-derived component that contains platelet concentrations above the normal level and includes platelet-related growth factors and plasma-derived fibrinogen [185,186]. Platelets are the frontline healing response to injuries, as they release growth factors (e.g., TGF-β and PDGF), cytokines (e.g., IL-1 and IL-6), and adhesion molecules (e.g., fibrin, fibronectin, vitronectin) essential for healing processes such as cell proliferation, angiogenesis, and ECM formation for tissue repair [187,188]. Clinically, PRP has been investigated as a treatment for OA and offers several advantages: it is autologous, relatively safe, easy to prepare, and associated with minimal side effects [189,190]. The IA PRP injections deliver a concentrated dose of these molecules directly to the affected joint, thereby promoting cartilage regeneration and reducing inflammation [177,180]. The treatment primarily targets cartilage and synovium, enhancing extracellular matrix synthesis and modulating synovial inflammation, ultimately improving cartilage integrity and alleviating joint pain [190,191].

An important step in the therapeutic use of PRP is its activation, which triggers platelets to release their growth factors. Activation is commonly achieved by administering calcium chloride and/or thrombin [64]. Once activated, PRP causes the platelets to degranulate, with nearly 100% of the growth factors being released within one hour of activation [192]. A recent meta-analysis demonstrated that exogenously activated PRP is more effective in improving pain and function than non-activated PRP in patients with knee OA [190,193]. However, conflicting evidence suggesting less efficient wound healing has raised questions about whether the rapid delivery of growth factors is ideal [186,188]. It remains unclear whether PRP should be activated, and since its efficacy can vary due to differences in preparation protocols, its regenerative capacity is generally more limited compared to stem cell-based therapies [190]. Despite these limitations, PRP is frequently studied in combination with stem cells to enhance therapeutic outcomes. Continued research is needed to standardize preparation methods and validate their long-term clinical effectiveness [194].

MSCs are multipotent stromal cells capable of differentiating into cartilage-producing chondrocytes [195,196,197]. These cells are gaining attention as potential treatments for OA due to their ability to target both cartilage and subchondral bone, contributing to structural repair and reducing chronic inflammation [195,197,198]. Clinical studies suggest that MSC therapy can improve joint function and delay disease progression, although outcomes vary depending on cell source, dose, and delivery method [199,200].

MSCs, derived from bone marrow, adipose tissue, or umbilical cord, exhibit immunomodulatory and chondrogenic properties [201]. These cells can differentiate into chondrocytes, secrete anti-inflammatory cytokines, and inhibit matrix-degrading enzymes [201]. Notably, MSC-derived exosomes, which are nano-sized extracellular vesicles rich in bioactive molecules, have been shown to reduce pro-inflammatory cytokines and modulate the NF-κB signaling pathway [183,184,185,186]. Additionally, bone marrow-derived MSCs (BMSCs) have been shown to enhance chondrocyte survival and suppress COX-2 expression, thereby limiting inflammatory pain and structural damage [187]. Moreover, various biomaterials such as hydrogels, scaffolds, and nanofibers have been engineered to support MSC survival, promote chondrogenesis, and enable the sustained release of bioactive compounds [202,203]. The synergy between MSCs and biomaterial scaffolds is being explored to enhance cartilage regeneration and restore joint function in preclinical and early clinical studies [203].

### 5.2. Gene Therapies for OA

Emerging gene therapy strategies aim to modify the expression of pro-inflammatory cytokines and catabolic enzymes involved in OA progression [204]. Viral vectors such as adeno-associated viruses and lentiviruses have been explored to deliver therapeutic genes directly into the joint, targeting molecules like IL-1β, TNF-α, MMP-13, and ADAMTS. For example, IL-1 receptor antagonist (IL-1Ra) gene delivery has shown promise in reducing joint inflammation and cartilage damage in preclinical models [205]. More recently, CRISPR-Cas9-mediated editing has been investigated to selectively disrupt catabolic gene pathways or enhance anabolic factors such as SOX9 and aggrecan [206]. While gene therapy holds great potential for disease modification, challenges remain in achieving targeted, sustained expression with minimal immunogenicity and off-target effects [207].

### 5.3. Disease-Modifying Osteoarthritis Drugs (DMOADs)

Unlike conventional treatments that focus on symptom relief, DMOADs aim to alter the structural progression of OA [208]. Several DMOAD candidates are currently being evaluated in clinical trials for their potential to delay or reverse structural progression in OA Table 2). The outcomes of these trials will determine the future landscape of OA therapeutics, especially for interventions that move beyond symptom control toward structural modification and long-term disease management [209]. While no DMOAD has yet received full regulatory approval, these agents represent a shift toward mechanism-based interventions aimed at modifying the disease trajectory rather than simply alleviating pain.

**Table 2:** Emerging pharmacologic and biologic disease-modifying therapies for OA and their molecular targets.

Treatment	Mode of Action	Target	Benefits
MMP-inhibitor PG-116800 (NCT01919164)	Inhibits cartilage matrix degradation	Cartilage matrix	Limits degradation and slows disease progression
Sprifermin (truncated FGF18)	Stimulates chondrocyte proliferation	Cartilage matrix	Improves cartilage thickness [210]
BMP-7 or OP-1 (NCT01133613, NCT01111045, NCT00456157)	Promotes chondrogenic differentiation	Cartilage matrix	Enhances cartilage repair and reduces pain [205]
AMG 108 (IL-1R1 antibody) (NCT00110942)	Inhibits IL-1β activity	IL-1 receptor	Reduces inflammation and failed to demonstrate significant clinical benefit [211]
Adalimumab (TNF inhibitor) (ACTRN 12612000791831)	Blocks TNF-α signaling	TNF-α receptor	Reduces pain and improves physical function
Infliximab	Inhibits TNF-alpha	TNFα receptor	Reduced progression of hand OA in recent-onset RA patients [212]
Tanezumab (anti-NGF antibody)	Blocks NGF-TrkA interaction	Targets NGF	Improves joint functional and pain scores, safety concerns, and NCT02697773
Trans-capsaicin (CNTX-4975)	Inhibits TRPV1 receptor	TRPV1	Decreases pain perception
Mavatrep (JNJ-39439335)	Inhibits TRPV1	TRPV1	Significant pain reduction but dose adjustments needed (EudraCT 2009-010961-21)
Selective agonist CR845	Inhibits opioid receptors	Activates kappa-opioid receptor	Dose-dependent pain reduction, effective in hip OA (NCT02524197 and NCT02944448)

Additionally, several approved drugs are being investigated as repurposed agents in the treatment of OA, such as liraglutide (anti-diabetic and anti-obesity drug: NCT02905864), Metformin (anti-diabetic drug: NCT04767841 and NCT05034029), and Zoledronic acid (anti-osteoporotic drug: NCT04303026) [164].

In summary, orthobiologic therapies, including PRP, MSC-based cell therapy, and tissue-engineering strategies, offer promising avenues for managing OA-related pain by targeting both inflammatory and degenerative processes within the joint [213,214]. Approaches range from intra-articular injections of bioactive molecules that reduce inflammation and modulate the joint environment to the transplantation of stem cells and biomaterials that promote cartilage repair [64,215]. While many of these therapies are still under investigation, early results indicate their potential to delay OA progression and alleviate chronic pain. Tissue engineering in OA aims not only to regenerate damaged tissues but also to reduce reliance on pharmacologic pain management and delay or eliminate the need for joint replacement.

## 6. Pain Measurement for Drug Development

Given the complexity of OA pain mechanisms, targeting the pain pathway for treatment is challenging. Various methods are employed to classify and measure evoked pain behaviors, weight-bearing deficits, gait abnormalities, and spontaneous pain behaviors in animals (Table 3). These include assessing mechanical allodynia in the hind paw using von Frey monofilaments, knee hyperalgesia through force application, thermal hypersensitivity on a hot/cold plate, static weight-bearing using a capacitance meter, dynamic weight-bearing through a specialized apparatus, gait analysis using the CatWalk or TreadScan systems, and monitoring spontaneous pain behaviors using video recording, conditioned place preference chambers, and a custom-made burrowing device [215,216,217,218,219].

Moreover, a comprehensive range of assessment tools is utilized for clinical evaluation for joint-related symptoms, function, patient perception, and activity levels. Several of these tools are summarized in Table 4 [196,197,198,199,200,201,202,203,204].

## 7. Conclusions

Growing evidence suggests that OA pain is not solely driven by structural joint damage but by a complex interplay between chronic low-grade inflammation, joint tissue degeneration, and alterations in pain processing pathways [21]. Inflammatory mediators sensitize peripheral nociceptors, while ongoing input from damaged joints can lead to central sensitization, amplifying the pain experience [248,249]. As a result, OA pain may persist despite minimal visible joint damage, often becoming chronic and difficult to treat with conventional therapies [250]. As our understanding of OA pathophysiology evolves, current research prioritizes the multi-targeted, mechanism-based treatments that address the interconnected biological and sensory drivers of disease [9]. This highlights the need for new treatment strategies that go beyond pain relief and focus on both inflammation and nerve-related pain, especially for patients who are not responsive to traditional pain medication [251].

Current nanomedicine platforms in clinical trials offer promising advancements in targeted and sustained IA drug delivery by localized therapeutic action with reduced systemic side effects and the direct modulation of the joint environment [252]. However, recent studies have emphasized the biological limitations of IA delivery, including rapid clearance and restricted tissue penetration, highlighting the need for integrative strategies that combine local and systemic therapies [253,254]. Therefore, IA drug delivery should be viewed not as a standalone solution but as one component of a comprehensive, patient-centered treatment plan [255]. Effective OA management must be comprehensive and patient-centered, combining IA therapies with systemic pharmacological agents, biomechanical interventions (e.g., physiotherapy and orthotics), and lifestyle modifications such as weight management and physical activity [254]. Incorporating these complementary approaches can enhance the therapeutic benefit of IA therapies, support long-term joint function, and improve overall patient outcomes [253,254].

## Figures and Tables

**Table 1:** Current OA treatments and their therapeutic targets.

Current OA Treatments	Mode of Action	Target	Potential Therapeutic Benefits
Corticosteroid Drugs (CSDs)	Reduction in inflammation and pain	Inflammatory mediators	Short-term relief of pain and inflammation
NSAIDs (Ibuprofen; Diclofenac)	Inhibit COX-1 and -2 enzymes	COX enzymes	Pain relief and reduced inflammation
Hyaluronic AcidIntraarticular Injections	Lubrication and pain reduction	Synovial fluid	Improved joint mobility and symptom relief
Tanezumab	NGF inhibition	Nerve growth factor	Reduction in pain and inflammation in OA
Cannabinoids	Activates CB1 and CB2 receptors	Cannabinoid receptors	Pain reduction and anti-inflammatory effects
Knee ReplacementSurgery	Surgical intervention for functional restoration	Knee joint	Long-term pain relief and functional improvement

**Table 3:** Pain assessment in animal models.

Pain Assessment	Species Used	Measurement/ Observation	Relevance to Human OA Pain
**Mechanical or Thermal Sensitivity**			
Von Frey Filaments [217,220]	rats; mice	Mechanical allodynia in hind paw using calibrated filaments	pain hypersensitivity seen in chronic OA
Pressure Application Measurement (PAM) [220]	rats	Knee hyperalgesia induced by applying controlled force to the joint until withdrawal or vocalization occurs	mechanical joint pain
Thermal Withdrawal Test [220]	rats; mice	Latency to withdraw from heat/cold stimuli	altered pain thresholds in OA
**Behavioral Assessment**			
Open Field Test [221]	rats; mice	Spontaneous locomotor activity and rearing	reduced mobility and pain-related behavioral changes
Conditioned Place Preference (CPP) [222,223]	rats; mice	Relief of spontaneous pain by linking the environment with analgesia	affective dimension of pain in humans
Burrowing Behavior Test [223]	rats; mice	Changes in innate behavior using a custom-made burrowing device	spontaneous pain
**Affective/Mimetic Assessment**			
Facial Grimace Scale [217]	mice, rats, sheep	Facial expressions (e.g., orbital tightening) to assess spontaneous pain	chronic human OA pain
Video Monitoring [224]	rats; mice	Continuous video tracking of spontaneous behaviors like licking, guarding, or rearing	spontaneous pain behaviors
**Functional Assessment**			
Static Weight-Bearing Test [225]	rats; mice	Unequal weight distribution using a limb loading capacitance meter	indicates joint discomfort and mechanical pain
Dynamic Weight-Bearing Test [226]	rats; mice	Voluntary limb loading during movement	mimics activity-related OA pain
Gait Analysis (CatWalk and TreadScan) [224]	rats, mice, Guinea pigs	Stride length, swing speed, and stance time using automated gait systems	reflects mobility impairments similar to human OA
Hind Limb Grip Strength [227]	rats	Limb muscle strength and coordination through grip meter	indirectly reflects joint discomfort or weakness

**Table 4:** Medical assessment of OA pain in humans.

Tools	Common Use Case	Measurement/ Observation
**Self-Report Tools**		
Numeric Rating Scale (NRS) [228,229]	General clinical use (acute/chronic pain)	Rates pain from 0 (no pain) to 10 (worst pain)
Visual Analog Scale (VAS) [229,230]	10 cm line for marking perceived pain level	Postoperative pain; research trials
Verbal Descriptor Scale (VDS) [229,231]	Elderly, cognitively impaired patients	Uses words (e.g., mild, moderate, severe) to describe pain
McGill Pain Questionnaire (MPQ) [232,233]	Neuropathic pain; chronic conditions	Evaluates sensory, affective, and evaluative qualities of pain
Brief Pain Inventory (BPI) [234]	Chronic pain; cancer pain	Assesses pain severity and functional interference
Pain DETECT Questionnaire [235,236]	Neuropathic pain screening	Screens for neuropathic pain characteristics
DN4 Questionnaire [237]	Neuropathic pain diagnosis	Checklist combining sensory symptoms and clinical signs
Knee Injury and Osteoarthritis Outcome Score (KOOS) [238]	Common in knee OA trials, especially post-surgery	Includes a pain subscale with items related to specific joint-loading activities
Hip Disability and Osteoarthritis Outcome Score (HOOS) [238]	Used in clinical studies and outcome assessments for hip OA	Adapted from KOOS; includes hip-specific pain and function items
SF-36 Bodily Pain Subscale [239]	Applied in OA studies to assess overall well-being	Part of a general health survey measuring pain and overall health-related QoL
WOMAC Pain Subscale [240]	Gold standard in OA clinical trials and interventions	Part of the Western Ontario and McMaster Universities Osteoarthritis Index; evaluates pain during five activities (e.g., walking and using stairs)
International Knee Documentation Committee (IKDC) Subjective Knee Evaluation Form [241]	Knee ligament and cartilage injuries	Pain, stiffness, swelling, and function during daily and sports activities
Cincinnati Knee Rating [242]	Knee injuries in athletes	Physician and patient ratings of pain, swelling, and functional activity
Lysholm Knee Scoring [243]	Post-ACL or meniscal	Knee function, including pain, instability, and stair climbing
Tegner Activity Scale [243]	Physical activity level	Activity levels from sedentary (level 0) to elite (level 10)
**Observational Tools**		
FLACC Scale [244]	Infants, ICU, non-verbal adults	Rates pain based on face, legs, activity, cry, consolability (0–10)
Facial Grimace Scale [245]	Neonates, unconscious, dementia patients	Evaluates facial expressions for spontaneous pain cues
**Experimental/Physiological Tools**		
Conditioned Pain Modulation (CPM) [246]	Central sensitization research	Measures descending inhibitory control through dual-pain paradigm
Quantitative Sensory Testing (QST) [247]	Neuropathic pain; sensory profiling	Measures mechanical and thermal thresholds to stimuli

## Data Availability

Not applicable.

## Abbreviations

The following abbreviations are used in this manuscript:

OA	Osteoarthritis
ECM	Extracellular Matrix
DRG	Dorsal Root Ganglion
ADAMTS	A Disintegrin and Metalloproteinase with Thrombospondin Motifs
DAMPs	Damage-Associated Molecular Patterns
IL1β	Interleukin 1β
IL1R1	Interleukin 1 Receptor 1
TNF-α	Tumour Necrosis Factor-alpha
TrkA	Tropomyosin receptor kinase A
LTβ	Lymphotoxin β
TLRs	Toll-like receptors
NF-κB	Nuclear Factor kappa-light-chain-enhancer of activated B cells
NGF	Nerve Growth Factor
JAK/STAT	Janus Kinase/Signal Transducer and Activator of Transcription
MAPK	Mitogen-Activated Protein Kinase
MMP	Matrix Metalloproteinase
MSCs	Mesenchymal Stem Cells
BMSCs	Bone Marrow-derived Mesenchymal Stem Cells
PRP	Platelet-Rich Plasma
PRRs	Pattern-Recognition Receptors
S100A8/9	S100 Calcium Binding Proteins A8/A9
IL-1Ra	IL-1 receptor antagonist
HMGB1	High-Mobility Group Box 1
HSPs	Heat Shock Proteins
PDGF	Platelet-Derived Growth
TGF-β	Transforming Growth Factor Beta
HS	Histamine
SP	Substance P
CSD	Corticosteroid Drugs
NRS	Numeric Rating Scale
VAS	Visual Analog Scale
VDS	Verbal Descriptor Scale
MPQ	McGill Pain Questionnaire
BPI	Brief Pain Inventory
KOOS	Knee Injury and Osteoarthritis Outcome Score
HOOS	Hip Disability and Osteoarthritis Outcome Score
IKDC	International Knee Documentation Committee
CPM	Conditioned Pain Modulation
QST	Quantitative Sensory Testing
